# Effects of Deletion of the *Streptococcus pneumoniae* Lipoprotein Diacylglyceryl Transferase Gene *lgt* on ABC Transporter Function and on Growth *In Vivo*

**DOI:** 10.1371/journal.pone.0041393

**Published:** 2012-07-24

**Authors:** Suneeta Chimalapati, Jonathan M. Cohen, Emilie Camberlein, Nathanael MacDonald, Claire Durmort, Thierry Vernet, Peter W. M. Hermans, Timothy Mitchell, Jeremy S. Brown

**Affiliations:** 1 Centre for Respiratory Research, Department of Medicine, Royal Free and University College Medical School, Rayne Institute, London, United Kingdom; 2 Infectious Diseases & Microbiology Unit, UCL Institute of Child Health, London, United Kingdom; 3 CEA, Institut de Biologie Structurale Jean-Pierre Ebel, Grenoble, France; 4 CNRS, Institut de Biologie Structurale Jean-Pierre Ebel, Grenoble, France; 5 Université Joseph Fourier – Grenoble 1, Institut de Biologie Structurale Jean-Pierre Ebel, Grenoble, France; 6 Laboratory of Pediatric Infectious Diseases, Radboud University Nijmegen Medical Centre, Nijmegen, The Netherlands; 7 Division of Infection and Immunity, IBLS, University of Glasgow, Glasgow, United Kingdom; Université Paris Descartes; INSERM, U1002., France

## Abstract

Lipoproteins are an important class of surface associated proteins that have diverse roles and frequently are involved in the virulence of bacterial pathogens. As prolipoproteins are attached to the cell membrane by a single enzyme, prolipoprotein diacylglyceryl transferase (Lgt), deletion of the corresponding gene potentially allows the characterisation of the overall importance of lipoproteins for specific bacterial functions. We have used a Δ*lgt* mutant strain of *Streptococcus pneumoniae* to investigate the effects of loss of lipoprotein attachment on cation acquisition, growth in media containing specific carbon sources, and virulence in different infection models. Immunoblots of triton X-114 extracts, flow cytometry and immuno-fluorescence microscopy confirmed the Δ*lgt* mutant had markedly reduced lipoprotein expression on the cell surface. The Δ*lgt* mutant had reduced growth in cation depleted medium, increased sensitivity to oxidative stress, reduced zinc uptake, and reduced intracellular levels of several cations. Doubling time of the Δ*lgt* mutant was also increased slightly when grown in medium with glucose, raffinose and maltotriose as sole carbon sources. These multiple defects in cation and sugar ABC transporter function for the Δ*lgt* mutant were associated with only slightly delayed growth in complete medium. However the Δ*lgt* mutant had significantly reduced growth in blood or bronchoalveolar lavage fluid and a marked impairment in virulence in mouse models of nasopharyngeal colonisation, sepsis and pneumonia. These data suggest that for *S. pneumoniae* loss of surface localisation of lipoproteins has widespread effects on ABC transporter functions that collectively prevent the Δ*lgt* mutant from establishing invasive infection.

## Introduction

Lipoproteins are a major category of bacterial surface proteins that have diverse functions, and often have important effects on pathogen/host interactions during the development of infection. The majority of bacterial lipoproteins are substrate-binding proteins for ABC transporters involved in the transport of a wide range of substrates including cations, sugars, aminoacids, oligopeptides, polyamines, and minerals and which individually can be vital for full virulence [1]–[6]. As well as their important role for bacterial physiology, lipoproteins are also key mediators of the inflammatory response to Gram positive pathogens through recognition by toll-like receptor 2 (TLR2) [7]–[9]. The mechanism of lipoprotein attachment to the bacterial cell membrane and processing is conserved amongst bacteria. After initial extracellular secretion of prolipoproteins by the general secretory pathway (directed by an N terminal signal peptide sequence), lipoproteins are covalently linked to the cell membrane by the enzyme diacylglyceryl transferase (Lgt) [10]–[12]. A type II lipoprotein signal peptidase (Lsp) then cleaves the N terminal signal peptide adjacent to the ‘lipobox’ cysteine residue to form the mature lipoprotein [12]–[14]. Loss of Lgt reduces the quantity of lipoproteins attached to the bacterial cell membrane and usually but not always prevents Lsp function [10], [15], [16].

The importance of individual lipoprotein components of ABC transporters for bacterial physiology would suggest that deletion of *lgt* should have profound effects on bacterial growth and survival. For Gram negative bacteria this seems to be the case, as mutation of *lgt* is fatal [17], [18]. In contrast, for a variety of Gram positive bacteria mutation of *lgt* does not prevent viability and often has surprisingly little effects on growth. For example the *lgt* mutants of *Streptococcus pneumoniae*, *Staphylococcus aureus*, *Streptococcus agalactiae*, *Streptococcus mutans*, *Streptococcus equi*, *Streptococcus suis, Streptococcus sanguinis,* and *Listeria monocytogenes* have similar or only mildly impaired growth compared to the parental wild-type strain in complete or rich media [9], [16], [19]–[26]. Growth of *lgt* mutants is more often impaired in restrictive media, with for example, reduced growth in tissue culture or iron deficient media for a *S. aureus lgt* mutant [16], [27] and poor growth of a *S. mutans lgt* mutant in medium containing only meliobiose as a carbon source [24]. Mutation of individual lipoproteins can also have effects on bacterial sensitivity to environmental stress, adhesion to host tissues, and interactions with host phagocytes [28]–[30]. Phenotypes that might reflect these lipoprotein dependent functions have been described for some *lgt* mutants, including reduced intracellular replication and increased sensitivity to cationic peptides for the *L. monocytogenes lgt* mutant [25], and reduced adhesion and resistance to oxidative stress for the *S. agalactiae lgt* mutant [19].

The effects of *lgt* mutation on virulence are also often surprisingly weak and variable between different bacterial pathogens. For example, Petit et al. have described a *S. pneumoniae lgt* mutant that has greatly reduced virulence in a mouse model of pneumonia, whereas other streptococcal *lgt* mutants have either only mildly impaired, normal or even in the case of *S. agalactiae* increased virulence (attributed to reduced TLR2 dependent inflammatory responses) [21]–[23]. At present there has only been limited characterization of the physiological consequences of loss of Lgt for streptococci, and so there is no explanation for why effects on virulence are so variable between species. The *S. pneumoniae* genome contains approximately 40 genes predicted to encode lipoproteins [31], [32], many of which are involved in virulence as part of nutrient uptake ABC transporters [1]–[3], [33]–[42]. In particular, cation ABC transporters have major effects on the ability of *S. pneumoniae* to cause infection, with loss of the PspA manganese transporter lipoprotein or combined loss of the AdcA and AdcAII zinc or the PiaA and PiuA iron ABC transporter lipoproteins all resulting in strains that are greatly reduced in virulence [2], [3], [36], [39], [40], [42]. Hence if loss of lipoprotein anchoring to the cell membrane impairs cation uptake this could readily explain the reduced virulence of the *S. pneumoniae lgt* mutant, but at present there are no data on the effects of loss of Lgt on ABC transporter functions for *S. pneumoniae*. In addition, the *S. pneumoniae* genome contains seven ABC transporters annotated as involved in sugar uptake, including probable raffinose, galactose, and maltose/maltodextrin transporters as well as transporters of uncharacterised sugar substrates [31]. Several publications suggest that ABC sugar transporters are also required for full virulence in mouse models of infection [1], [33], [43]. However their importance might be offset by the considerable potential for redundancy in sugar acquisition due to the presence of multiple phosphoenolpyruvate (PEP)-dependent phosphotransferase system (PTS) sugar transporters in the *S. pneumoniae* genome. Assessing the effects of the *lgt* mutation on growth in different sugars could identify whether ABC transporters are necessary for *S. pneumoniae* sugar uptake despite the presence of numerous putative PTS sugar transporters.

We have therefore investigated whether a *S. pneumoniae* Δ*lgt* mutant has phenotypes related to impaired cation and/or carbohydrate acquisition and the consequences of the *lgt* mutation for growth in physiological fluids. *S. pneumoniae* commonly causes infections in the blood as well as the lung, and colonises the nasopharynx. The physiological conditions at these varied sites vary substantially and this could affect the relative importance of lipoprotein function for bacterial survival. Hence, we have also investigated the effects of the *lgt* mutation on *S. pneumoniae* infection at these different anatomical sites.

## Results

### The lgt Operon and Construction of a Δ*lgt S. pneumoniae* Strain

**Figure 1:**
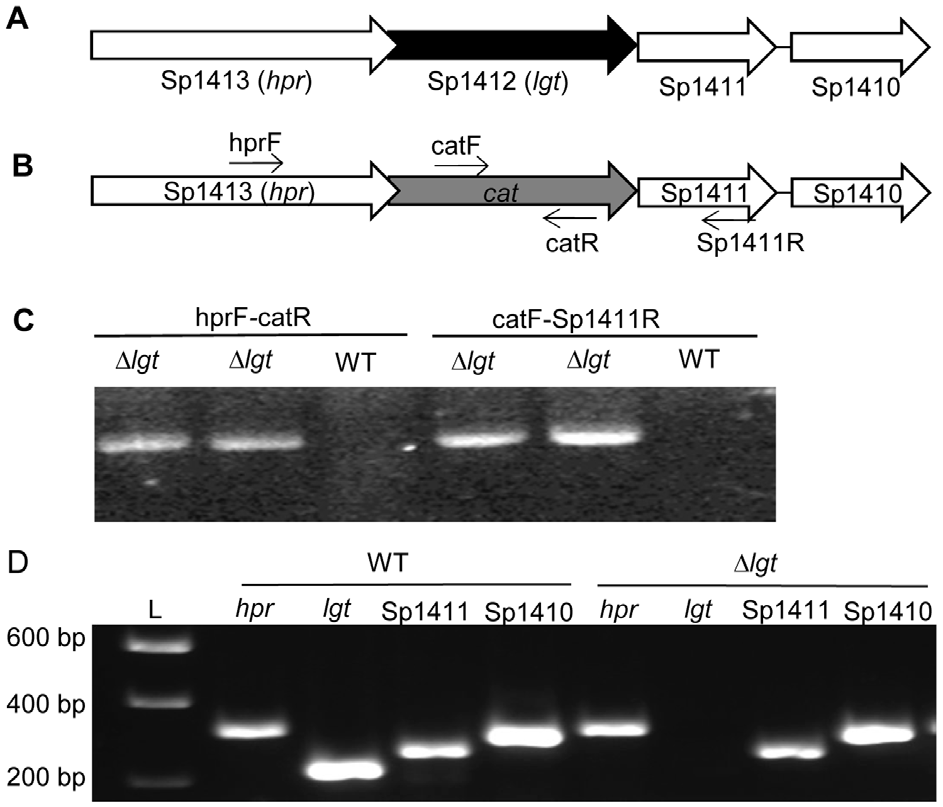
Construction of the Δ*lgt* strain. (A) Schematic of the Sp_1410–1413 locus, with the TIGR4 genome gene number and the assigned gene names in parentheses when available. Arrows indicate transcriptional direction and *lgt* is shaded black. (B) Structure of the Sp_1410–1413 locus in the Δ*lgt* mutant strain, showing replacement of *lgt* with an in-frame copy of *cat* which is shaded grey and position of primers used in (C). (C) Gel red stained agarose gels showing PCR analysis of two separately obtained Δ*lgt* strains confirming replacement of *lgt* with *cat*. Primer pairs (Table 4) and the strain used for each reaction are given above each lane. (D) Gel red stained agarose gels of RT-PCR reactions using internal primers (Table 4) for each gene within the putative Sp_1410–1413 operon, confirming the non-polar deletion of *lgt*. Reactions not containing reverse transcriptase gave no products (not shown), and L marks the DNA ladder size marker with sizes listed on the left.

In the *S. pneumoniae* TIGR4 genome, the gene encoding Lgt is Sp_1412, the second gene in a putative four gene operon with either overlapping or very closely spaced open reading frames (Fig. 1A). The predicted product of *lgt* has a high degree of identity and similarity to the Lgt of other bacteria (Table 1). The other genes in this operon encode an Hpr (ser) kinase/phosphatase (Sp_1413) and two hypothetical proteins with unknown function (Sp_1411 and Sp_1410) (Fig. 1). BLAST searches show that homologs of Sp_1413 are associated with *lgt* in several other Gram positive bacteria, including *S. suis*, *Streptococcus pyogenes*, *S. aureus*, and *Lactococcus lactis*. To study the role of Lgt in *S. pneumoniae*, a non-polar deletion mutant (Δ*lgt*) was created in which the Sp_1412 gene was replaced in frame by a chloramphenicol resistance cassette (*cat*) (Fig. 1B). Non-polar deletion of *lgt* was confirmed by PCR (Fig. 1C) and reverse transcriptase PCR (RT-PCR), which demonstrated the continued transcription of the remaining genes in this putative operon (Fig. 1D). The stability of the Δ*lgt* mutant was confirmed by growth in THY without added chloramphenicol for two consecutive growth cycles and then plating on to blood agar plates with and without chloramphenicol, which resulted in 100% recovery of chloramphenicol resistant bacteria. Despite multiple attempts including insertion of an intact copy of *lgt* within the Sp_1413-10 operon or ectopically (data not shown) we have been unable to create a genetically complemented Δ*lgt* strain.

**Table 1:** Blast alignments of the Sp1412 (Lgt) amino acid sequence to other organisms.

Organism	Gene number	Size (No of amino acids)	% identity/similarity^a^
*S. sanguinis*	HMPREF8578_1725	262	89/94 (202)
*S. suis*	SSU05_1605	267	68/83 (266)
*S. agalactiae*	SAL_0792	257	65/83 (192)
*S. equi*	Sez_1357	259	63/89 (199)
*S. mutans*	SmuNN2025_1248	263	65/80 (197)
*L. monocytogenes*	LMFG_00890	277	56/70 (205)
*Bacillus subtilus*	BSU6633_04292	269	53/69 (179)
*S. aureus*	SALG_00828	279	49/65 (176)
*Escherichia coli*	ECO157_010100032601	291	28/48 (185)

a Length of the amino acids compared.

### Lipoprotein Localisation in the *S. pneumoniae* Δ*lgt* Strain

**Figure 2:**
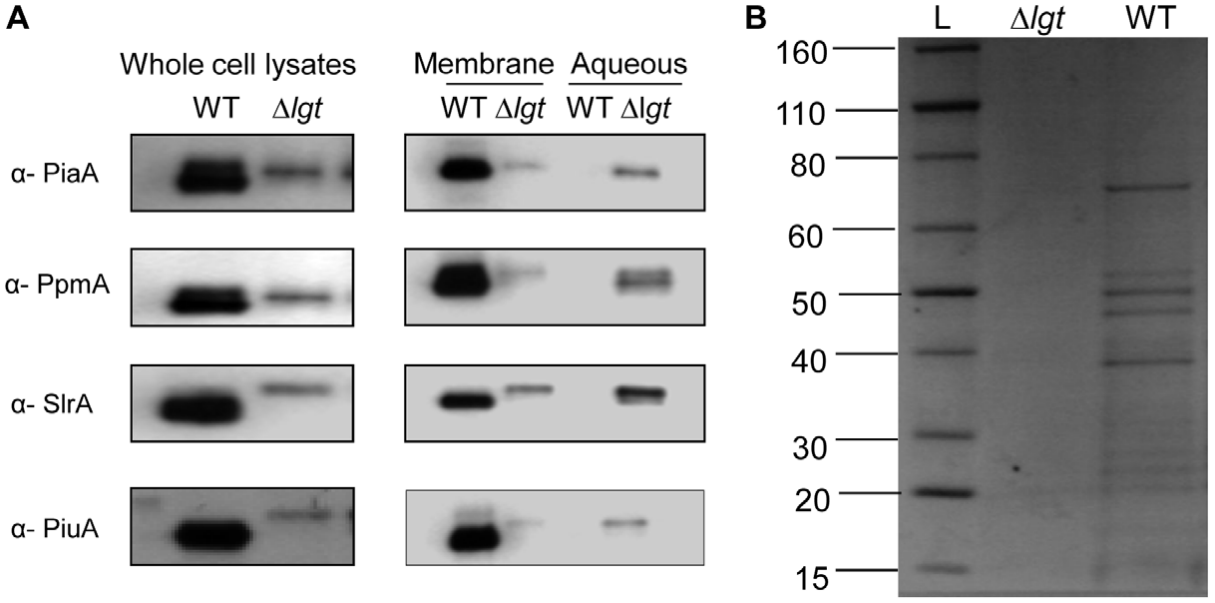
Effects of the Δ*lgt* mutation on the localisation of *S. pneumoniae* lipoproteins. (A) Immunoblots of whole cell lysates and the membrane and aqueous phases of triton X-114 extracts of wild-type (WT), Δ*lgt* strains using antibodies to the *S. pneumoniae* lipoproteins PiaA, PpmA, SlrA and PiuA. (B) Coomasie blue staining of triton X-114 extracted membrane lipoproteins Δ*lgt* and wild-type (WT) strains when separated on SDS–PAGE. Lane L, a standard protein ladder with molecular weights ranging from 15 KDa To 80 KDa.

The effect of *lgt* deletion on *S. pneumoniae* lipoproteins was assessed by immunoblots of whole cell lysates using polyclonal mouse or rabbit antibodies to four *S. pneumoniae* lipoproteins, the iron ABC transporter lipoproteins PiuA and PiaA [2], and the non-ABC transporter associated lipoproteins PpmA and SlrA (kind gift from Peter Hermans, Radboud University) [44]. Although equal amounts of protein were loaded for both strains, the signal for all the lipoproteins investigated was stronger in the wild-type strain (Fig. 2A, lane 1) compared to the Δ*lgt* strain, indicating reduced abundance of lipoproteins in the Δ*lgt* strain. Membrane-associated proteins from the wild-type and the Δ*lgt* strains were extracted using triton X-114, a non-ionic detergent which solubilises and extracts lipidated membrane proteins into the detergent phase with hydrophilic proteins remaining in the aqueous phase [45]. Immunoblots for the lipoproteins in the triton X-114 and aqueous extracts revealed a strong signal in the triton X-114 fraction for the wild-type strain with no detectable signal in the aqueous fraction (Fig. 2A), confirming that the lipoproteins in the wild-type are localised to the cell membrane. In contrast, for the Δ*lgt* strain the signal for all the lipoproteins investigated was much weaker in the triton X-114 fraction and significant quantities of the lipoproteins were found in the aqueous fraction (Fig. 2A). Coomassie brilliant blue staining of the SDS-PAGE gel of the triton X-114 extracted proteins from the wild type strain demonstrated a large number of protein bands ranging between 15 and 80 KDa which previously we have shown to represent a range of lipoproteins including the cation transporters PiaA, AdcA and PsaA, and potential sugar transporters MalX and Sp_1683 [44]. However, these bands were largely absent for the triton X-114 extract from the Δ*lgt* strain (Fig. 2B). These data indicate that, as expected, deletion of *lgt* resulted in loss of a number of lipoproteins from the membrane in the Δ*lgt* strain including cation and sugar transporters.

**Figure 3:**
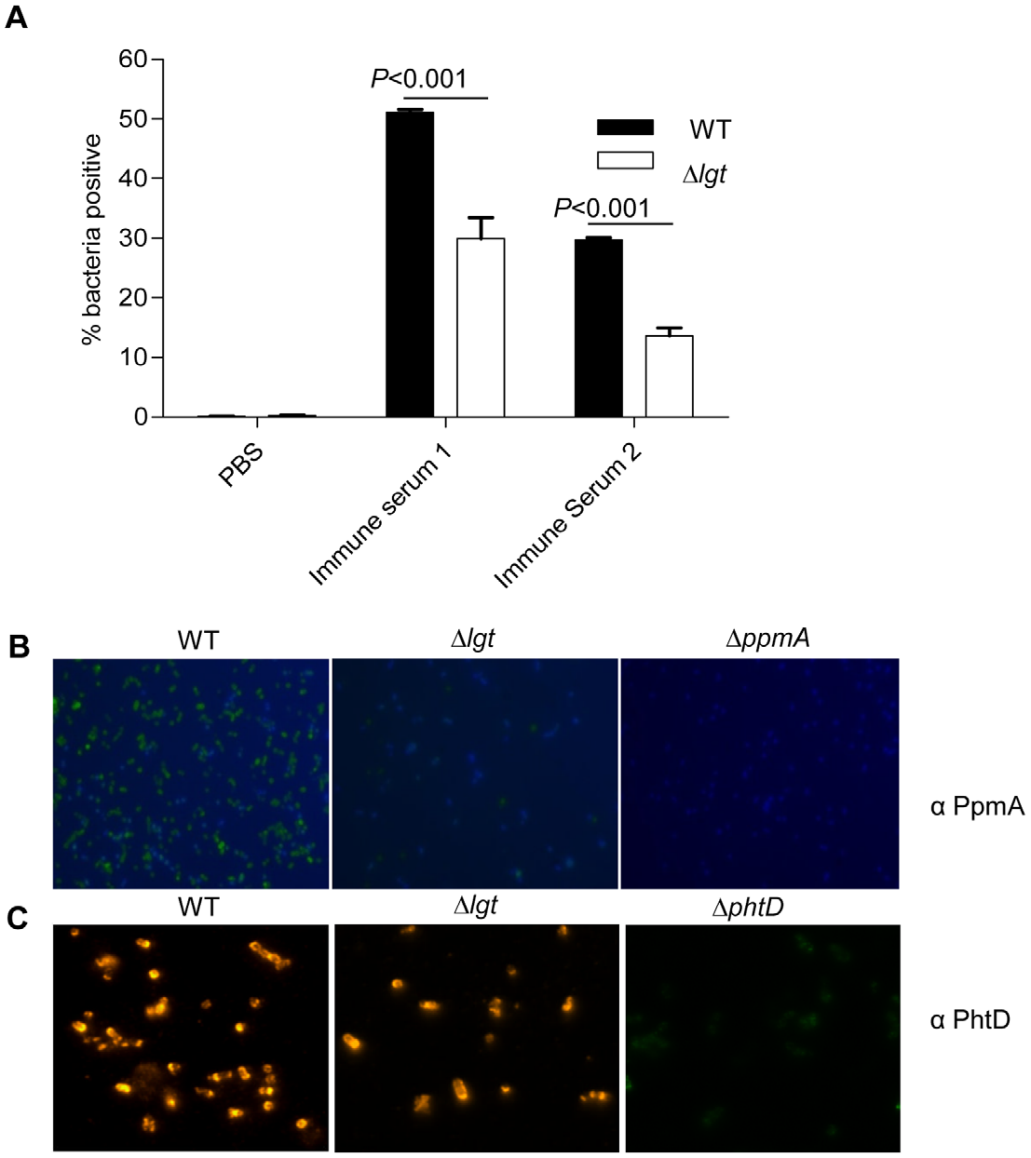
Effect of the Δ*lgt* mutation on surface accessability of *S. pneumoniae* lipoproteins. (A) Flow cytometry analysis of the mean proportion of bacteria positive for IgG binding after incubation with immune sera containing high antibody titres towards lipoproteins of *S. pneumoniae*. Black columns represent wild-type strain and clear columns represent the Δ*lgt* strain. Error bars represent SDs and *P* values were obtained using multiple ANOVA test with post-hoc analysis. (B) Immunofluorescence of the *S. pneumoniae* wild-type (WT), Δ*lgt* and Δ*PpmA* strains using anti- PpmA antibody and FITC conjugated secondary antibody. (C) Immunofluorescence of the *S. pneumoniae* wild-type (WT), Δ*lgt* and Δ*PhtD* strains using anti-PhtD antibody and Cy2 conjugated secondary antibody.

To further confirm the reduced cell surface location of lipoproteins in the Δ*lgt* strain, IgG binding to live *S. pneumoniae* wild-type and Δ*lgt* strain bacteria after incubation in polyclonal mouse sera from mice vaccinated with a *S. pneumoniae* Δ*pab* strain [46] was assessed using flow cytometry. This sera contains high IgG antibody titres to the lipoproteins PsaA and PpmA as well several non-lipoprotein antigens [46]. IgG binding to the Δ*lgt* strain was significantly reduced compared to IgG binding to the wild-type strain, compatible with reduced IgG recognition of lipoproteins in the Δ*lgt* strain due to their loss from the bacterial surface (Fig. 3A). Furthermore, immuno-fluorescence microscopy using polyclonal antibodies to PpmA identified significant fluorescence with wild-type *S. pneumoniae* but much reduced fluorescence for the Δ*lgt* strain and no fluorescence for the negative control Δ*ppmA* strain (Fig. 3B). In contrast, immunoflorescence microscopy using polyclonal antibodies to the cell wall protein PhtD was not affected by in the Δ*lgt* strain (Fig. 3C). Taken together, the immunoblots of triton X-114 extracts, flow cytometry and immuno-fluorescence microscopy demonstrate that the quantity of lipoproteins localised to the cell membrane and available for interactions with external agents is greatly reduced in the Δ*lgt* strain.

### Cation ABC Transporter Function in the Δ*lgt* Strain

**Figure 4:**
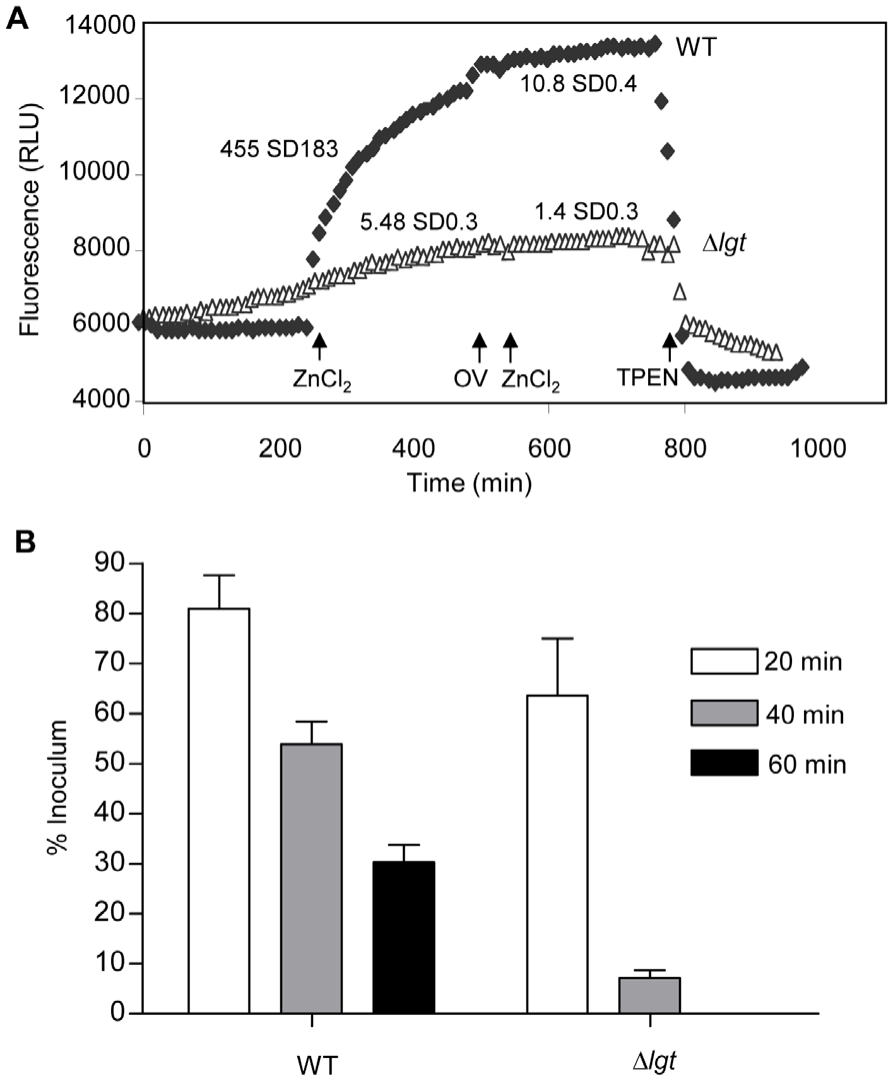
Cation dependent phenotypes of the Δ*lgt* strain. (A) Uptake of Zn^+2^ by the Δ*lgt* (triangles) and wild-type (filled diamonds) strains measured using a using FluoZin-3 fluorescence. The arrows numbered 1, 2 and 3 indicate the time points at which ZnCl_2_, 1 mM ortho-vanadate and TPEN were added to the strains respectively. Mean (SD) Zn^2+^ uptake in RFLU sec^−1^ before and after addition of orthovanadate and 10 µm ZnCl_2_ are stated next to the corresponding line. For comparison of zinc uptake by wild-type and Δ*lgt* strains, *P* = 0.01 using Student’s t-test. (B) Proportion of wild-type and Δ*lgt* strain bacteria surviving after exposure to 60 mM paraquat for 20 min (clear columns), 40 min (grey columns) and 60 min (black columns). No Δ*lgt* strain bacteria survived after 60 minutes incubation. For the comparison between wild-type and Δ*lgt* strains, at 40 and 60 min time points *P* values were <0.01 and <0.05 (2 way ANOVA with post-hoc tests).

The ABC transporters Adc and AdcAII are required for zinc uptake by *S. pneumoniae* [39], [40]. Hence to directly assess the effects of the *lgt* mutation on a cation ABC transporter, zinc uptake was quantified using the fluorescent probe FluoZin-3 which fluoresces with an excitation/emission wavelength of 495/516 nm respectively when intracellular concentrations of zinc increase [42], [47]. After the addition of 10 µM ZnSO_4_, the wild-type strain showed a steady increase in fluorescence with time whereas there was only a minimal increase in fluorescence of the Δ*lgt* strain (Fig. 4A). The rate of Zn^+2^ uptake, calculated from the slope of the curve was markedly lower in the Δ*lgt* strain compared to that of the wild-type (Fig. 4A). After addition of a further 10 µM ZnSO_4_ preceded by 1 mM orthovanadate, an ATPase inhibitor [48], there was no further Zn^+2^ uptake even in the wild-type strain, confirming that the uptake of Zn^+2^ was ABC transporter mediated. The specificity of the FluoZin-3 assay for Zn^2+^ was confirmed by the addition of TPEN, a high affinity, membrane permeable Zn^2+^ chelator, which resulted in quenching of the fluorescence response in wild-type bacteria (Fig. 4A). These data demonstrate that the reduced membrane localisation of lipoproteins in the Δ*lgt* strain was associated with markedly reduced function of zinc uptake ABC transporters.

To investigate whether the Δ*lgt* strain had significant difficulties in obtaining other cations imported using ABC transporters, intracellular cations concentrations were measured using ICP-MS (Table 2). For the Δ*lgt* strain intracellular concentrations of Fe^2+^, Zn^2+^, Mn^2+^, Ni^2+^ and Cu^2+^ were all significantly reduced, with values ranging from less than 1/100 (Fe^2+^ and Ni^2+^) to 1/9 (Mn^2+^) of the values obtained for the wild-type strain. Intracellular Mn^2+^ imported by the lipoprotein PsaA is required by *S. pneumoniae* to protect against oxidative stress [30], [49]. Hence, to help confirm a reduced cation content of the Δ*lgt* strain, the sensitivity of the wild-type and the Δ*lgt* strains to oxidative stress was assessed using 60 mM paraquat. Only 7% (SD 2.3) of the Δ*lgt* strain inoculum remained viable after 20 min incubation with paraquat compared to the 53.9% (SD 6.37) of the wild-type strain, and after 60 minutes no Δ*lgt* strain bacteria were recovered compared to 30.3% (SD 4.8) of the wild-type strains (Fig. 4B). Overall, the results of these assays demonstrate that the Δ*lgt* strain has a phenotype compatible with the defective function of several cation ABC transporters.

**Table 2:** Quantification of intracellular *S. pneumoniae* and media cation contents using ICP-MS and expressed in Ppb (+/− SD).

Cation	*S. pneumoniae* strain		Media		
	Wild-type	Δ*lgt*	THY	THY Chelex	C+Y
Fe^2+^	3800+/−38	32+/−2	NA	NA	NA
Mn^2+^	18+/−1	1.9+/−0.1	670+/−4	1.31+/−0.02	5.2+/−0.2
Zn^2+^	1020+/−22	37+/−4.3	2237+/−45	17.52+/−3.14	241+/−34
Ni^2+^	110+/−1	0.8+/−0.02	520+/−50	0.45+/−0.03	6.2+/−0.3
Cu^2+^	380+/−4	16+/−0.5	920+/−11	1.02+/−0.17	11.4+/−4

NA – not available.

### Effects of Limited Cation Availability on Growth of the *S.* *pneumoniae* Δ*lgt* Strain

**Figure 5:**
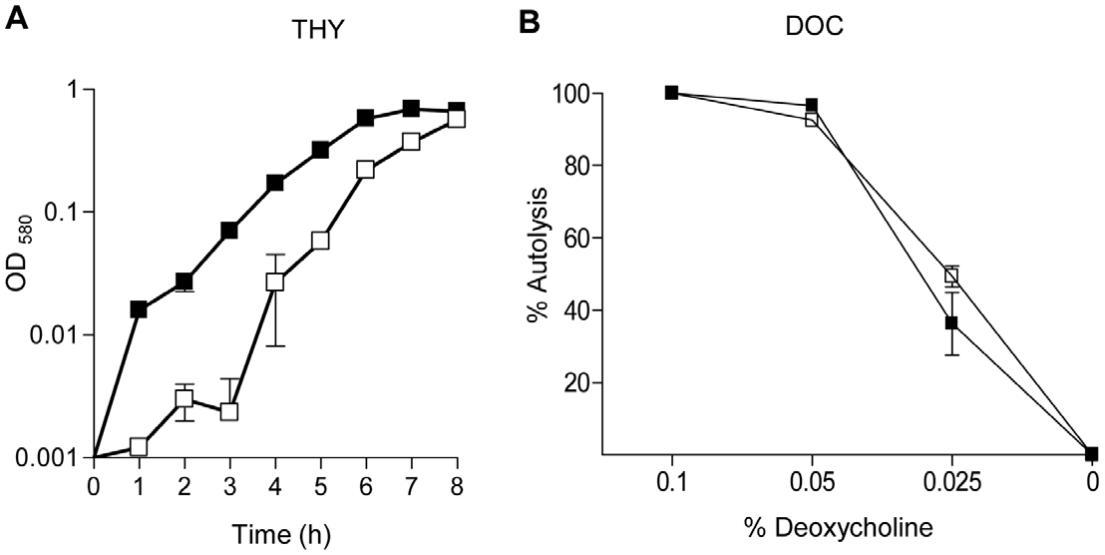
Growth of the wild-type and Δ*lgt* strains in complete medium and susceptibility to DOC-induced lysis. (A) Growth of the wild-type and Δ*lgt* strains in THY assessed by measuring broth culture log_10_ OD_580_ over time. (B) Proportion of bacteria surviving after incubation with increasing concentrations of DOC. Squares represent the wild-type strain, triangles the Δ*lgt* strain. Error bars represent SDs, and when not visible are within the symbol.

**Figure 6:**
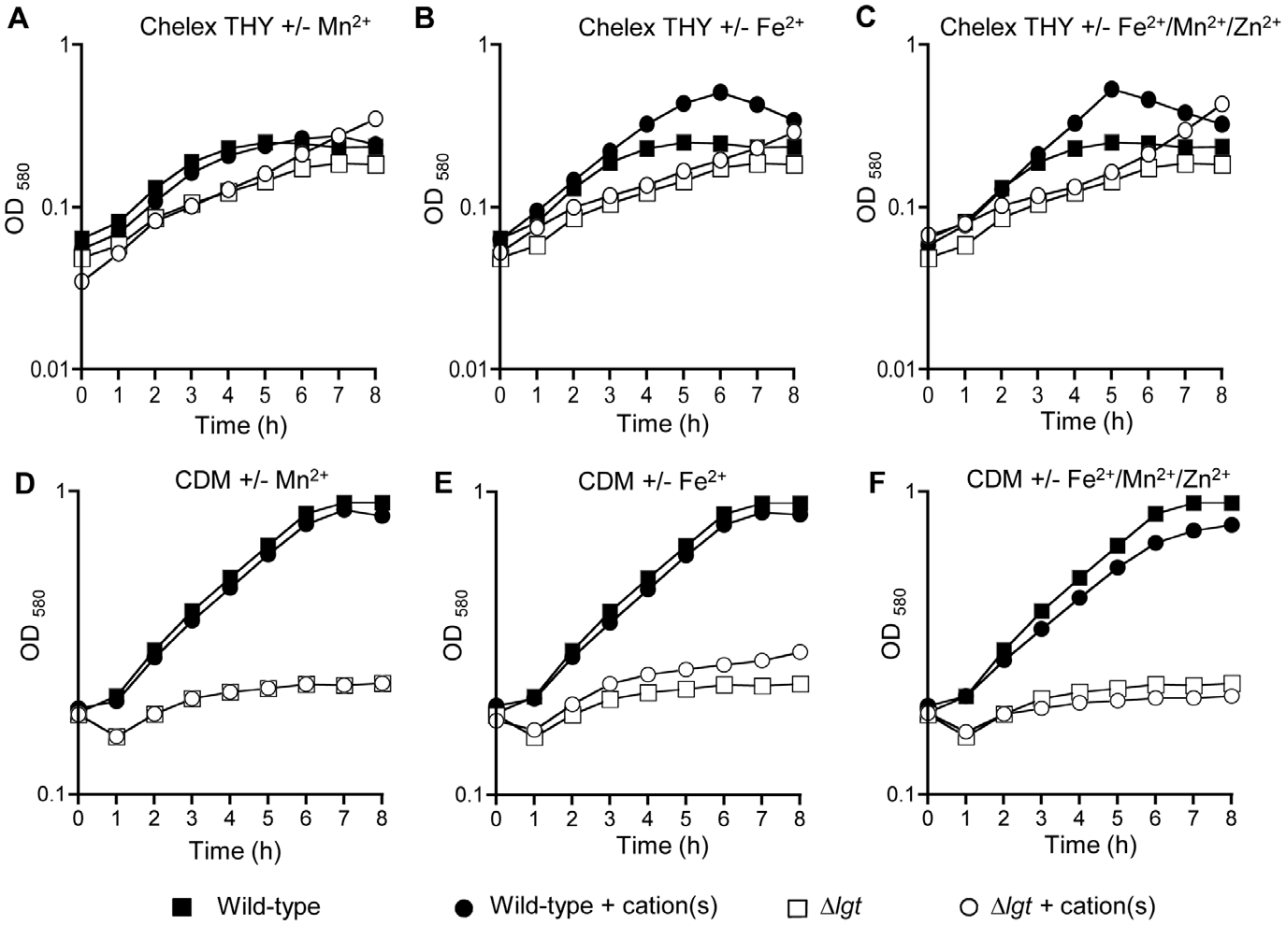
Growth of the wild-type and Δ*lgt* strains in cation depleted media. Assessed by measuring broth culture log_10_ OD_580_ over time. (A) to (C) Growth in cation depleted chelex-THY and (D) to (E) in CDM with and without cation supplementation: (A) and (D) with and without Mn^2+^ supplementation; (C) and (E) with and without Fe^2+^ supplementation; and (D) and (F) with and without combined Mn^2+^, Fe^2+^ and Zn^2+^ supplementation. Filled symbols represent growth of the wild-type strain, empty symbols growth of the Δ*lgt* strain. Squares represent growth in unsupplemented media, inverted triangles in media supplemented with 50 µM Mn^2+^, diamonds in media supplemented with 50 µM Fe^+2^ and circles in media supplemented with 50 µM Mn^+2^, Fe^+2^ and Zn^+2^.

To investigate the physiological consequences of impaired cation transport, growth of the Δ*lgt* and wild-type strains was compared in the complete medium THY, in THY treated with chelex to deplete cation availability, and in chemically defined media with known concentrations of cations. Although there were no significant difference in the doubling times between the wild-type and the Δ*lgt* strain in THY (Table 3), the Δ*lgt* strain did have a longer lag phase (Fig. 5A) demonstrating that the Δ*lgt* strain had some growth defect even in this undefined complete medium. The Δ*lgt* strain was also very slightly more susceptible to lysis in response to increasing concentrations of deoxycholate (DOC) (Fig. 5B). In chelex-THY the Δ*lgt* strain had a markedly increased doubling time and reduced maximum OD_580_ compared to the wild-type strain (Table 3, Fig. 6A). Supplementation of chelex-THY with Zn^2+^ impaired growth of both the wild-type and Δ*lgt* strains (Table 3), compatible with the known toxicity of excess zinc to *S. pneumoniae* [50]. Supplementation of chelex-THY with Mn^2+^ had little effect on growth of the wild–type strain but decreased the doubling time of the Δ*lgt* strain and allowed it to eventually reach a maximum OD_580_ similar to the wild-type strain, suggesting a reduced ability to acquire Mn^2+^ is one cause of the reduced growth of the Δ*lgt* strain in chelex-THY (Table 3, Fig. 6A). Supplementation of chelex-THY with Fe^2+^ markedly enhanced the maximum OD_580_ reached by the wild-type strain, indicating as previously demonstrated that lack of iron is the major limiting factor for the growth of this strain in chelex-THY [51] (Table 3, Fig. 6B). For the Δ*lgt* strain supplementation with Fe^2+^ had a small effect on the maximum OD_580_ but no effect on the doubling time, suggesting the Δ*lgt* strain was unable to fully utilise exogenous iron to overcome the growth defect caused by treating THY with chelex. Supplementation with all three cations enhanced growth of the wild-type strain no more than supplementation with Fe^2+^ alone, but for the Δ*lgt* strain increased the maximum OD_580_ to a greater extent than supplementation with Fe^2+^ or Mn^2+^ alone (Table 3, Fig. 6C). These data suggest an impaired ability to obtain Mn^2+^ and Fe^2+^ by the *S. pneumoniae* Δ*lgt* strain could cause growth defects in cation restricted conditions. Growth of the Δ*lgt* strain was very poor in CDM media even when supplemented with all three cations (Fig. 6D to F) preventing the assessment of the effects of specific nutrient deficiencies using this media.

**Table 3:** Doubling times (mins) (SD)^a^ for the wild-type and Δ*lgt* strains in different media.

Broth medium	Wild-type	Δ*lgt*	Ratio Δ*lgt/*wild-type
THY	45.0 (3.6)	44.7 (2.82)	0.99
C+Y+ glucose^a^/sucrose^b^	49.3 (2.44)	59.4 (1.56)	1.20
C+Y+ glucose^a^	47.8 (3.79)	63.0 (1.14)	1.32
C+Y+ raffinose^c^	63.0 (4.85)	77.0 (1.35)	1.22
C+Y+ maltotriose^b^	57.3 (4.56)	83.5 (2.55)	1.46
Chelex THY	92.4 (5.24)	244.6 (8.23)	2.65
Chelex THY +Fe^2+^	96.7 (4.25)	259.9 (6.58)	2.69
Chelex THY +Mn^2+^	94.5 (3.45)	173.2 (8.65)	1.83
Chelex THY +Zn^2+^	106.6 (5.68)	n/c	–
Chelex THY +Fe^2+^+Mn^2+^+Zn^2+^	96.7 (3.68)	166.3 (4.88)	1.72

n/c = not calculated as the slope of increase of OD_580_ was too shallow for an accurate assessment of the doubling time.

a = uptake PTS system dependent.

a = uptake ABC transporter and PTS system dependent.

c = uptake ABC transporter dependent only.

### Effects of Limited Carbohydrate Sources on Growth of the *S. pneumoniae* Δ*lgt* Strain

**Figure 7:**
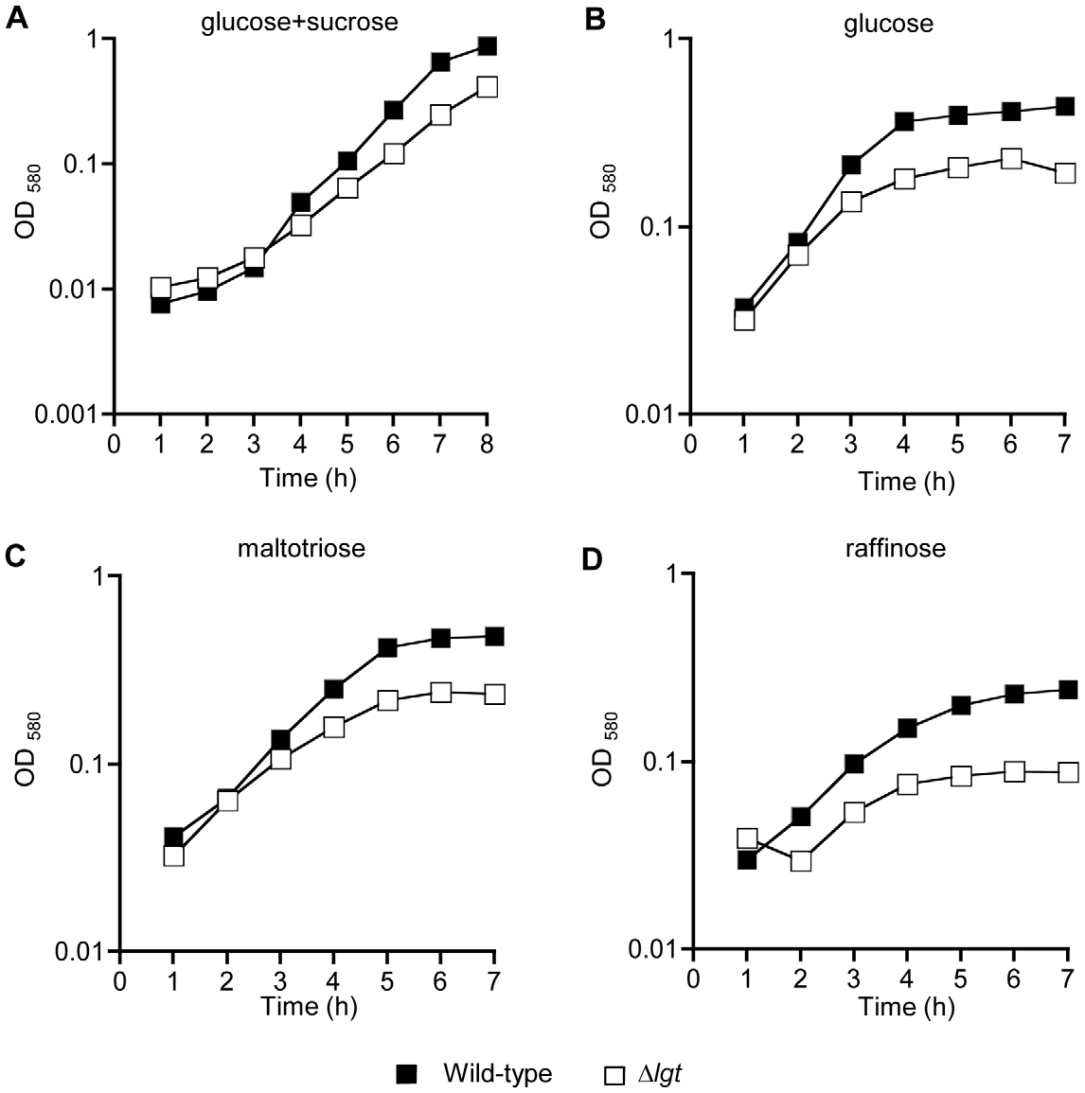
Growth of the wild-type and Δ*lgt* strains in restricted carbohydrate sources. Assessed by measuring broth culture log_10_ OD_580_ over time. (A) C+Y medium supplemented with 10 mM sucrose and glucose each; (B) C+Y medium supplemented with 10 mM glucose; (C) C+Y medium supplemented with 10 mM maltotriose; and (D) C+Y medium supplemented with 10 mM raffinose. Error bars represent SDs, and when not visible are within the symbol. Filled symbols represent growth of the wild-type strain, empty symbols growth of the Δ*lgt* strain.

In the *S. pneumoniae* genome seven ABC transporters are annotated as involved in sugar uptake, including probable raffinose, galactose, and maltose/maltodextrin transporters but excluding a glucose transporter [31], [52]. Of these only raffinose is imported by an ABC transporter system alone, with import of the other sugars also occurring via by at least one PTS system [52]. The global reduction of lipoproteins in the Δ*lgt* strain allowed the investigation of whether sugar ABC transporters are vital for growth in conditions with restricted carbon sources or whether PTS transporters provide adequate sugar uptake. The growth of the Δ*lgt* strain was compared to the wild-type strain in the partially defined cation supplemented medium C+Y containing specific sugars as the sole carbohydrate source. Compared to the wild-type strain growth of the Δ*lgt* strain was slightly delayed when sucrose and glucose in combination were the sole carbohydrate source similar to the growth results for THY (Table 3 and Fig. 7A). When glucose, raffinose, or maltotriose were the sole carbohydrate sources the impaired growth of the Δ*lgt* strain compared to the wild-type was increased and a lower maximum OD_580_ achieved, with the most marked affect seen when raffinose was the sole carbohydrate source (Fig. 7B–D). There were also slight increases in the ratio of the doubling times for the wild-type and Δ*lgt* strains in C+Y with glucose or maltotriose (Table 3). These data indicate that loss of lipoproteins significantly impaired growth of *S. pneumoniae* in restricted carbohydrate sources, despite the potential for PTS systems to compensate for reduced ABC transporter function.

### Effects of lgt Deletion on Replication of *S. pneumoniae* in Physiological Fluids and Interactions with Neutrophils

**Figure 8:**
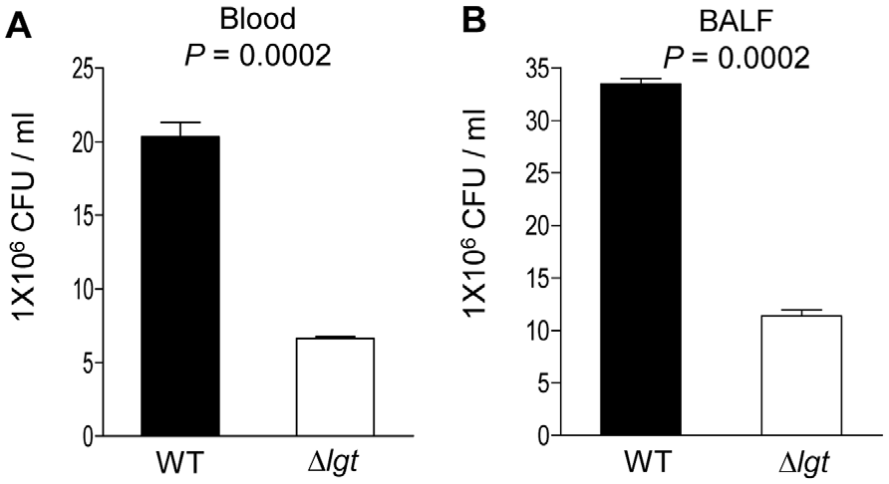
Growth of the wild-type and Δ*lgt* strains in blood (A), or BALF (B). (A) and (B) Bacterial CFU after 4 hours replication in blood (B) or BALF (B). Data is presented as the mean (SD) bacterial CFU per ml for the wild type (black columns) and the Δ*lgt* strain (clear columns). *P* values were obtained using two-tailed Student’s t-tests.

**Figure 9:**
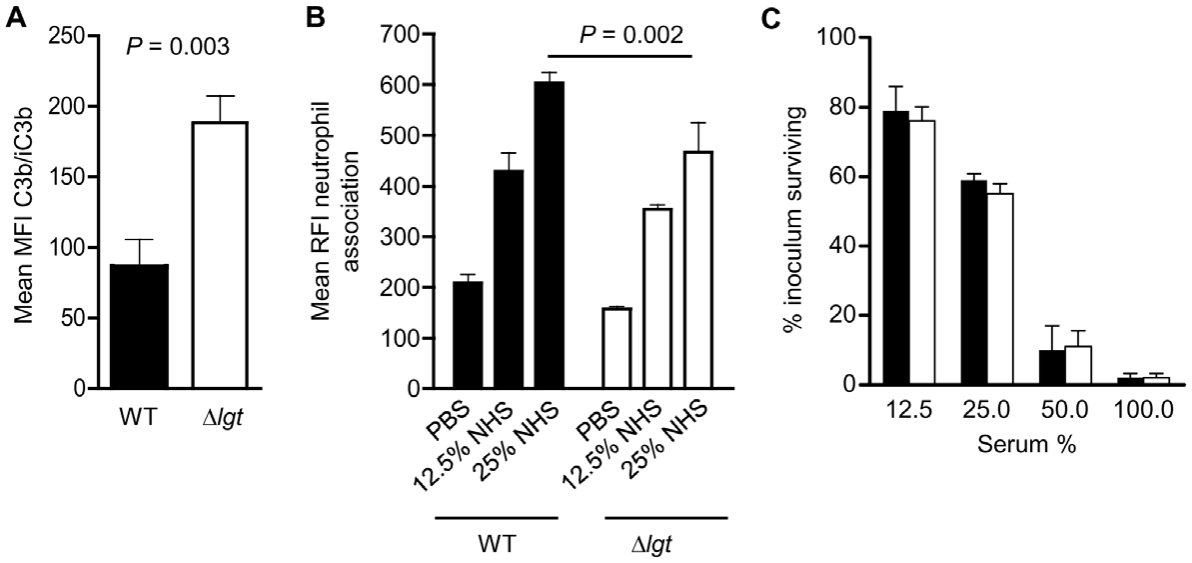
Effects of the Δ*lgt* mutation on interactions with neutrophils. (A) C3b/iC3b deposition on the wild-type and Δ*lgt* strains after incubation in 20% serum as measured by geometric mean fluorescent intensity using a flow cytometry assay. (B) Neutrophil association of the wild-type and Δ*lgt* strains after incubation in 20% serum and human neutrophils as measured by mean relative fluorescent intensity using a flow cytometry assay. (C) Neutrophil killing assays of the wild-type (black columns) and Δ*lgt* strains (clear columns) after incubation in different concentrations of human serum with fresh human neutrophils (MOI 1 bacteria to 800 neutrophils). There were no statistically significant differences between the wild-type and Δ*lgt* strains. Data are presented as the percentage of the inoculum surviving after 30 mins incubation. For all panels, data are for mean values with error bars representing SDs. Statistical comparisons were made using unpaired two tailed T tests, and *P* values inserted for selected data showing significant differences.

To investigate whether the effects of the *lgt* mutation on growth in restricted media results in impaired *S. pneumoniae* replication in physiologically relevant conditions, the replication rates of the wild-type and Δ*lgt* strains in human blood and mouse bronchoalveolar lavage fluid (BALF) were compared. In blood, after 4 hours incubation CFU of the wild-type strain had increased 5.1-fold whereas CFU of the Δ*lgt* strain had increased only 1.5-fold (Fig. 8A). The reduced increase in Δ*lgt* strain CFU could be caused by poor replication of this strain in blood or by increased sensitivity to neutrophil killing. Flow cytometry assays showed that complement deposition was increased on the Δ*lgt* strain compared to the wild-type, yet association with neutrophils (mainly due to phagocytosis) [53] was slightly lower (Fig. 9A and B). Overall, there were no differences seen between the wild-type and the Δ*lgt* strain in a neutrophil-killing assay (Fig. 9C). Furthermore the Δ*lgt* strain also replicated poorly in cell free BALF, with an increase in CFU of only 1.2-fold after 4 hours compared to 3.3-fold for the wild-type strain (Fig. 8B). These data suggest that the *lgt* mutant strain replicates poorly under physiological conditions and that the mutation has some effects on interactions with phagocytes without leading to major changes in bacterial susceptibility to neutrophil killing.

### Effect of lgt Deletion on Virulence of *S. pneumoniae*

**Figure 10:**
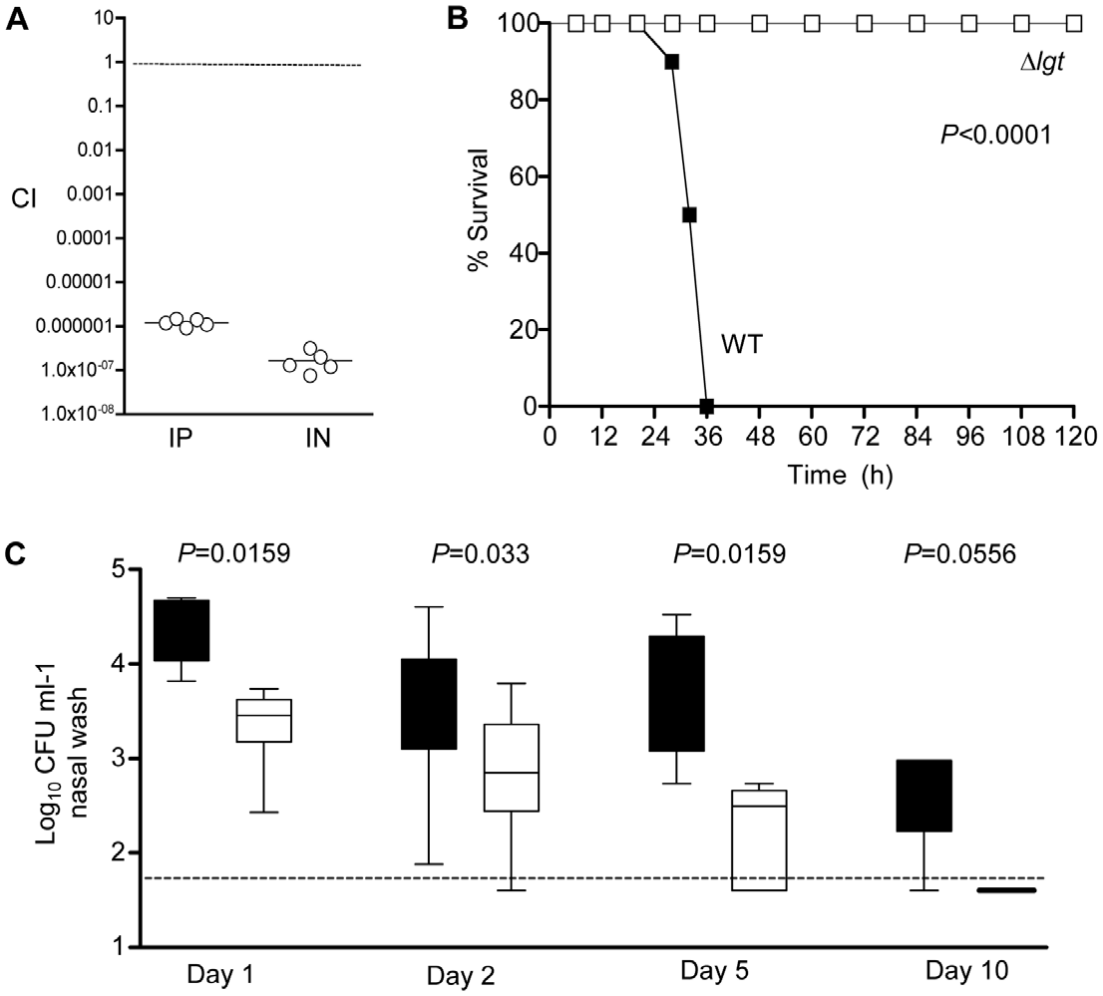
Virulence of the Δ*lgt* mutant strain. (A) CIs for the Δ*lgt* strain versus the wild-type strain in mouse models of septicaemia at 24 hours (IP inoculation, bacteria recovered from the spleen) and pneumonia at 48 hours (IN inoculation, bacteria recovered from the lungs). Each point represents the CI for a single animal. (B) Time course of the development of fatal infection for groups of 10 mice inoculated IP with 3×10^3^ CFU of the wild-type (WT) and Δ*lgt* strains (*P*<0.0001, log rank test). (C) Log_10_ bacterial CFU per ml of nasal wash recovered 1, 2, 5 and 10 days after nasopharyngeal colonisation of mice with 5×10^6^ CFU of the wild-type (black columns) and Δ*lgt* D39 (clear columns) strains. *P* values were calculated using Mann Whitney U tests for each time point.

Previously the *S. pneumoniae* Δ*lgt* strain has been shown to be impaired in virulence in a mouse model of pneumonia [23]. To investigate whether the virulence of the Δ*lgt* strain is also impaired during sepsis we initially used competitive infections. For both septicaemia and pneumonia models, after inoculation in a 50/50 ratio with the wild-type 0100993 strain no Δ*lgt* CFU were found in the spleen or lungs respectively despite recovery of >5 log wild-type CFU ml^−1^, giving CIs of less than 0.0001 (Fig. 10A). These data demonstrate that the Δ*lgt* strain had a severe competitive disadvantage during infection, but even very low CIs sometimes do not reflect an inability to cause infection when the mutant strain is given as a pure inoculum [1], [44]. Hence, to further investigate the degree of attenuation in virulence of the Δ*lgt* strain we used a mouse model of sepsis in which inoculation of 100 wild-type CFU is fatal. Groups of 10 mice were inoculated IP with 3×10^3^ CFU of the wild-type or Δ*lgt* 0100993 strain and the development of disease monitored over time. All mice inoculated with the wild-type strain developed fatal infection within 50 hours, whereas no mice infected with the Δ*lgt* strain showed signs of disease and all survived beyond 14 days (Fig. 10B). To assess the ability of the Δ*lgt* strain to establish infection in the lung, mice were inoculated IN with 5×10^6^ CFU of the wild-type or Δ*lgt* 0100993 strain and bacterial CFU calculated by serial plating of BALF recovered 4 hours later. For mice inoculated with the wild-type strain 4.3 log_10_ (SD 0.75) CFU ml^−1^ of BALF were recovered, whereas for the Δ*lgt* strain no CFU were recovered from any mice. These data confirm that the *lgt* mutant is avirulent during systemic infection and is very rapidly cleared from the lungs in the pneumonia model, compatible with the in vitro growth defects for the Δ*lgt* strain when cultured in blood or BALF. The physiological conditions in the nasopharynx are significantly different to those within the lung and the blood, and could potentially support growth of the *lgt* strain. Hence whether loss of lipoproteins prevented *S. pneumoniae* colonisation of the nasopharynx was investigated by transferring the Δ*lgt* mutation to the capsular serotype 2 D39 strain which (unlike the serotype 3 0100993 strain) can colonise the mouse nasopharynx for at least 11 days [54]–[56]. The D39 Δ*lgt* strain was able to establish colonisation of the nasopharynx for up to 5 days, demonstrating that this strain was still able to replicate at this anatomical site. However, the D39 Δ*lgt* strain was entirely cleared from the nasopharynx by day 10, at which time point the majority of mice were still colonised with wild-type D39 (Fig. 10C). Furthermore, there were approximately half a log_10_ CFU fewer present per ml of nasal wash compared to the results for the wild-type D39 strain at days 1, 2, and 5 (Fig. 10C). Hence loss of surface lipoproteins strongly impaired nasopharyngeal colonisation by *S. pneumoniae* as well as preventing systemic infection.

## Discussion

Lipoproteins are an important class of surface associated proteins that have diverse roles and frequently are involved in the virulence of bacterial pathogens. As lipoproteins are attached to the cell membrane by a single enzyme, Lgt, with additional processing by Lsp, deletion of the corresponding genes potentially allows the investigation of the global function of lipoproteins for an individual bacterial species. Several *lgt* mutants of Gram positive bacteria have been described, but the published data have shown that the phenotypes of *lgt* mutant strains vary with species. In particular, the *lgt* mutation has a strikingly pleiotropic effect on bacterial virulence, causing markedly reduced virulence for some species, no effect on virulence for other species, and even in some publications increasing the virulence of *S. aureus* and *S. agalactiae* [9], [22]. In contrast, the consequences of loss of Lgt for lipoprotein attachment are very similar between species, resulting in a greatly reduced lipoprotein content of the cell membrane for the *lgt* mutant strains [15], [16], [57]. Why *lgt* mutations vary in their associated phenotypes between species probably therefore reflect differences between bacterial species in the functional consequences of reduced lipoprotein content.

Previously, Petit et al. have demonstrated that in contrast to other streptococci a *S. pneumoniae* Δ*lgt* strain was greatly reduced in virulence in a mouse model of pneumonia [23]. The reasons for the loss of virulence of the *S. pneumoniae lgt* mutant were not characterised. We have confirmed the loss of virulence of the *S. pneumoniae* Δ*lgt* strain and demonstrated that this strain is also avirulent during systemic infection and is cleared from the lungs within 4 hours of inoculation. Multiple *S. pneumoniae* ABC transporters have significant roles during disease pathogenesis [33]–[35], including the manganese transporter Psa [36], the iron transporters Piu, Pia and Pit [3], [51], amino acid transporters [1], [37], the polyamine transporter Pot [38], the zinc transporters AdcA and AdcAII [39], [40], [42], and the phosphate transporter Pst [41]. We have therefore investigated the effects of the *lgt* mutation on ABC transporter related functions that might affect virulence, specifically concentrating on cation transport due to the profound effects of impaired manganese, iron or zinc uptake on *S. pneumoniae* virulence [36], [42], [51]. As expected, immunoblots, flow cytometry and immunofluorescence all showed a marked reduction in surface-associated lipoproteins for the *S. pneumoniae* Δ*lgt* strain and retention of the N terminal signal peptide, a similar phenotype to the Δ*lgt* mutants for most other bacteria [15], [16], [57]. The phenotype of the *S. pneumoniae* Δ*lgt* strain suggested this strain has impaired ability to acquire a range of cations, with markedly reduced Zn^2+^ uptake, an increased sensitivity to oxidative stress compatible with low Mn^2+^ levels, and an accentuated growth defect in cation-depleted medium. In addition the Δ*lgt* strain had greatly reduced intracellular levels of Fe^2+^, Mn^2+^, Zn^2+^, Cu^2+^ and Ni^2+^, cations that are either known to be or are predicted to be acquired by *S. pneumoniae* or other bacterial pathogens using ABC transporters [2], [3], [5], [36], [39], [58]. Hence, the Δ*lgt* strain has defects in acquisition of several cations that are known to affect virulence. Although we have been unable to complement the Δ*lgt* strain, RT-PCR confirmed continued transcription of the downstream genes in the *lgt* operon, and the multiple phenotypes of this strain compatible with impaired ABC transporter function are unlikely to be caused by an unidentified secondary mutation that occurred during the transformation process.

Reduced iron uptake is thought to partially explain reduced virulence of a *S. aureus lgt* mutant [27], and similarly reduced uptake of cations could readily explain why the *S. pneumoniae* Δ*lgt* strain cannot cause invasive infection. In addition, the effects of the *lgt* mutation on other ABC transporters could also be relevant. For example, growth curves also suggested the *S. pneumoniae* Δ*lgt* strain had impaired utilisation sugar sources. The largest difference in OD_580_ compared to the wild-type strain was seen when raffinose was the sole carbohydrate source, supporting recent data suggesting raffinose is the only sugar transported only by an ABC transporter system [52]. However the primary sugar available in blood is glucose, which is transported by a PTS systems alone [52]. Impaired uptake of other ABC transporter substrates such as phosphate, polyamines and amino acids could also cause reduced virulence, as might loss of function of non-ABC transporter lipoproteins such as PpmA and SlrA [1], [29], [38], [41], [59]. The main mechanisms of bacterial clearance during *S. pneumoniae* infection is neutrophil phagocytosis [60]. Although the increased sensitivity of the Δ*lgt* strain to oxidative stress might be assumed to result in increased susceptibility to neutrophil oxidative killing mechanisms, *S. pneumoniae* killing is independent of oxidative killing mechanisms [61], and mice with defects in oxidative killing are actually more resistant to *S. pneumoniae* infection [62]. Furthermore we have previously demonstrated that the effects of defects in resistance to oxidative killing on virulence were independent of oxidative killing mechanisms [63]. In vitro assays gave conflicting results about the susceptibility of the Δ*lgt* strain to opsonophagocytosis. This strain had some increased sensitivity to complement activity yet reduced uptake by neutrophils when incubated in human sera, possibly due to reduced lipoprotein targets for specific serum antibody [46]. Overall the Δ*lgt* strain did not have an increased susceptibility to neutrophil killing. Other important immune mechanisms such as anti-bacterial peptides or effects of the Δ*lgt* mutation on adhesion could potentially contribute to the reduced virulence of this strain. However, these immune mechanisms are mainly thought to be important during mucosal infection [60], [64] and are unlikely to cause such a severe virulence defect after intraperitoneal inoculation. Although increased susceptibility to host immunity may account for some of the loss of virulence, the data suggest loss of virulence of the *S. pneumoniae* Δ*lgt* strain was largely due to its major growth defects under physiological conditions as a consequence of impaired ABC transporter function. This was confirmed by demonstrating that the Δ*lgt* strain had a greatly reduced replication rate in blood or BALF compared to the wild-type parental strain.

Why does loss of Lgt has such a strong effect on the virulence of *S. pneumoniae* compared to the same mutation in other streptococci? Similar numbers of lipoproteins are expressed by *S. pneumoniae* as other streptococci (eg *S. agalactiae*), so it is not simply that there are more lipoproteins in *S. pneumoniae*. However, unlike the *S. pneumoniae* Δ*lgt* strain mutant the *S. agalactiae lgt* mutant had no growth defect in cation-depleted medium [19] and the *S. sanguinis lgt* mutant only had a growth defect in complete medium during competitive infection with the wild-type strain [20]. In addition, reduced zinc uptake has a more profound effect on *S. pneumoniae* virulence than for other bacterial pathogens [42], [65]–[67]. These data suggest that lipoprotein-dependent functions are generally of greater importance during *S. pneumoniae* infection than they are for other streptococci, resulting in a stronger phenotype for the Δ*lgt* mutant in animal models. Despite the profound effects on virulence during lung and systemic infection, the *S. pneumoniae* Δ*lgt* strain could colonise the nasopharynx for up to 5 days demonstrating lipoprotein functions are of lesser importance for bacterial replication in the nasopharyngeal environment compared to the lung or in the blood. This observation perhaps suggests that the acquisition of lipoprotein-dependent functions is one factor that allows *S. pneumoniae* to be an invasive pathogen.

Previously we have shown that deletion of the zinc uptake lipoproteins *adcA* and *adcAII* prevented nasopharyngeal colonisation by *S. pneumoniae* [42], a more profound defect than observed with the *S. pneumoniae* Δ*lgt* strain. In addition, despite the range of functions associated with ABC transporters and lipoproteins that together might be predicted to essential for bacterial viability, the *S. pneumoniae* Δ*lgt* strain still grew in complete and some restricted media as well as the mouse nasopharynx. This suggests that the partial retention of prolipoproteins on the surface of the *lgt* mutant shown by the immunoblots and immunofluorescence results in some functional activity. Alternatively uptake ABC transporters functions may have a residual level of function even without their lipoprotein component, but this seems unlikely given the profound phenotype of the *adcA* and *adcAII* double mutant [42]. Similarly for some phenotypes in *S. sanguinis*, *S. equi*, and *B. subtilus* deletion of a single lipoprotein had stronger effects than mutation of *lgt*, and this was thought to be due to partial lipid anchoring of prolipoproteins via the retained N terminal signal peptide [15], [20], [21]. For the *S. pneumoniae* Δ*lgt* strain some retention of prolipoprotein in the cell membrane may allow adequate ABC transporter function for growth under conditions with limited stress such as in complete medium or the nasopharynx. However, blood or the lungs are likely to be more stringent environments that require a greater level of lipoprotein function for sufficient *S. pneumoniae* replication to cause infection, resulting in loss of virulence of the Δ*lgt* strain. For many Gram positive pathogens lipoproteins are major ligands for TLR2-dependent inflammatory responses [8], [68], but their role during inflammatory responses to *S. pneumoniae* has not been evaluated as yet. The effects of lipoproteins on inflammatory responses need investigating as potentially compensatory TLR4 and TLR-independent mechanisms of inflammation during *S. pneumoniae* infection have been described, and data from animal models questions the overall importance of TLR2 during *S. pneumoniae* infection [69]–[73]. Even if lipoproteins are important pro-inflammatory signals during infection with *S. pneumoniae* and the *lgt* strain was able to avoid immune recognition, an inability to replicate during invasive infection would still prevent this strain from causing significant infection.

In conclusion, we have presented data demonstrating that deletion of the *S. pneumoniae lgt* results in a mutant strain with reduced cation uptake, increased sensitivity to cation and sugar restriction, and with poor growth in physiological media resulting in an inability to cause invasive infection. This striking contrast with the infection phenotypes of *lgt* mutants for some other bacterial pathogens suggest lipoprotein and ABC transporters have a corresponding greater importance during the development of infections caused by *S. pneumoniae* than they do for at least some other streptococci.

## Methods and Materials

### Ethics Statement

Experiments were approved by the UCL Biological Services Ethical Committee and the UK Home Office (Project Licence PPL70/6510). Experiments were performed according to UK national guidelines for animal use and care, under UK Home Office licence.

### Bacterial Strains and Culture Conditions

*S. pneumoniae* strains used in this work are listed in Table 4. The mutant strains used for this work were constructed in the 0100993 capsular serotype 3 clinical isolate [34]. *S. pneumoniae* strains were cultured at 37°C and 5% CO_2_ on Columbia agar supplemented with 5% horse blood, in Todd–Hewitt broth supplemented with 0.5% yeast extract (THY). chloramphenicol (10 µg ml^−1^) and erythromycin (0.2 µg ml^−1^) were added to blood agar plates where appropriate. Cations were depleted from the THY medium by treating it with 2% chelex-100 (Bio-Rad) overnight under continuous agitation and filtering the medium with 0.45 µ filters [44], [51]. Growth of strains was compared in broth culture by measuring OD_580_ in THY, THY-chelex, Chemically Defined Medium (CDM) [74] and a semi synthetic medium, C+Y [75] media at regular intervals. Working stocks of bacterial cultures in THY (OD_580_ 0.3–0.4) were stored at −80°C with 10% glycerol.

**Table 4:** Strains and primers used in this study.

Name	Description/sequence (source/reference)^a^
Strains	
0100993	*S. pneumoniae* capsular serotype 3 clinical isolate [34]
Δ*lgt* ST3	0100993 with in-frame deletion of Sp1412: cm^r^ (this study)
JSB3*PpmA* ^−^	0100993 with deletion of *PpmA* : ery^r^ [44]
D39	*S. pneumoniae* capsular serotype 2 strain (kind gift from James Paton, University of Adelaide)
Δ*lgt* D39	D39 with in-frame deletion of Sp1412: cm^r^ (this study)
Primers	
Sp1411F	GAGTCATCAAGAGCTTCGG
Cm-1411R	GCCTAATGACTGGCTTTTATAAATGTTAGAAGTTGCATATATTC
Cm-1413F	ACATTATCCATTAAAAATCAAATCAAGCAT TTTGCACCTCATTT
Sp1413R	CATGCCTTCCAACAGCCG
CmF	TTATAAAAGCCAGTCATTAG
CmR	TTTGATTTTTAATGGATAATG
hprRTF	GGTGACCACGTTTGACAAG
hprRTR	CTGATCAGCATGCCTTCC
lgtRTF	GGCCGTGATATGACCTCG
lgtRTR	GTTTGGCCATTTACGGTGG
Sp1411RTF	GCTGACAGACTTGCACCAG
Sp1411RTR	GCTTGGTCGTGTCATCGATG
Sp1410RTF	GAGTCATCAAGAGCTTCGG
SP1410RTR	GGTGCAGCTCTTGCCTTG

### Construction of Δ*lgt* Deletion Mutant Strain

For the in-frame deletion of lgt (Sp1412), a construct was created in which 703 bp of flanking DNA 5′ to the SP1412 ATG (primers Sp1413F and Cm-Sp1413R) and 750 bp of flanking DNA 3′ to the Sp1412 ORF (primers Cm-Sp1411F and Sp1411R) were amplified by PCR from *S. pneumoniae* 0100993 genomic DNA and fused with the chloramphenicol resistance marker (*cat*, amplified from pID701, a suicide vector containing *cat* gene, with primers CmF and CmR) by overlap extension PCR [76]. Primers used for the overlap extension PCRs are shown in Table 2. The constructs were transformed into *S. pneumoniae* by homologous recombination and allelic replacement using competence stimulating peptide (CSP-1) and standard protocols [34], [77].

### DNA, RNA Extraction and RT PCR

Genomic DNA and total RNA were isolated from *S. pneumoniae* strains using the Wizard genomic DNA isolation kit and the SV total RNA isolation system (Promega) respectively, following the manufacturer’s instructions except that cells were incubated with 0.1% deoxycholicacid (Sigma) at 37°C for 10 min before extraction. 0.5% RNasin (Promega) was added to extracted RNA to prevent it from degradation. cDNA was derived and amplified from RNA using the Access RT-PCR system (Promega) and target specific primers. Primers used for the transcriptional analysis of the Sp1410-1413 operon are described in Table 2. The National Centre for Biotechnology Information website (http://blast.ncbi.nlm.nih.gov/Blast) was used for DNA and protein BLAST searches.

### Protein Immunoblots and Triton X-114 Extraction

Protein samples from whole cell lysates and triton X-114 extracts were separated on SDS-PAGE 12% resolving gels, blotted onto nitrocellulose membranes and probed with specific antisera (1∶2500 dilution) according to standard protocols [78]. Membrane proteins were extracted by triton X-114 extraction as described previously [45], [79]. Briefly, exponentially growing *S. pneumoniae* cells were digested with 100 µl of 0.1% DOC (Sigma) in PBS for 30 min at 37°C and sonicated with 3 pulses of 15 sec with a 10 sec cooling time using a Soniprep 150 (Sanyo) ultrasonicator. 800 µl of PBS and 100 µl of triton X-114 (10% in PBS) were then added to the lysates, which were incubated at 4°C for 2 h followed by centrifugation to pellet insoluble debris. Supernatants were then incubated at 37°C for 30 min to allow phase separation, followed by centrifugation at room temperature to pellet the detergent phase proteins. The detergent phase proteins were washed and diluted 1∶2 in PBS prior to solubilization in Laemmli sample buffer for SDS-PAGE.

### IgG Binding to Live *S. pneumoniae* TIGR4

Flow cytometry assays of IgG deposition on the surface of *S. pneumoniae* strains were performed using a previously described protocol of Jomaa et al. [80] and mouse sera obtained from surviving mice after systemic infection with an attenuated TIGR4 mutant strain (unpublished data). Bacterial pellets containing 5×10^6^ CFU, pooled mouse serum (1∶5 dilution in PBS), and 1∶50 dilution of phycoerythrin conjugated goat anti-mouse IgG (Jackson ImmunoResearch) were used for the assay. Results are presented as the percentage of bacteria positive for IgG binding.

### Immunofluorescence Microscopy

The immunofluorescence microscopy was performed according to a previously described method [4]. Briefly, bacteria grown to an OD_580_ of 0.3 in THY broth were washed in PBS prior to fixing in 3% paraformaldehyde for 15 min at room temperature followed by 45 min on ice. Cells were then deposited onto poly-L-lysine-coated slides and permeabilized in cold methanol for 5 min. Slides were blocked for 30 min at room temperature with 5% (w/v) skimmed dry milk in PBS (saturation buffer) and then incubated for 1 h with anti-PpmA antibody (1∶50 dilution) in saturation buffer. The slides were then washed twice in PBS and incubated in the dark with a 1∶200 dilution of FITC-conjugated goat anti-rabbit immunoglobulin G (Jackson immunoresearch) in saturation buffer for 1 h. After successive washes with PBS and water, cells were incubated with ProLong gold mounting agent (Invitrogen, UK) containing 6-diamidino-2-phenylindole (DAPI) and dried overnight. The slides were examined with an Zeiss Axioscope microscope equipped with Zeiss Acroplan 100x O-PH/3 objective and a QImaging Retiga- SRV 1394 cooled charge-coupled device camera.

### C3b/iC3b Deposition and Neutrophil Phagocytosis Assays

To assess the effect of *lgt* deletion on the complement deposition on *S. pneumoniae* and on the interaction with phagocytes flow cytometry assays were performed according to previously described methods [46], [53]. For C3b/iC3b deposition 2×10^6^ CFU of bacterial pellets, human serum (1∶4 dilution in PBS) and FITC-conjugated polyclonal goat anti-human C3 antibody (ICN Cappel, Aurora, OH, USA, 1∶300) were used. The proportion of bacteria positive for C3b/iC3b and mean fluorescence intensity (MFI) was obtained using a FACSCalibur flow cytometer (Becton Dickinson, San Jose, USA), collecting data from at least 20,000 bacteria. For the opsonophagocytosis assay, the proportion of freshly extracted human neutrophils associated with 5, 6-carboxyfluorescein-succinimidyl ester (FAM-SE, Molecular Probes, Eugene, Oreg) labelled fluorescent bacteria (1×10^6^ CFU) was measured by flow cytometry after opsonization with 1/8 and 1/4 dilutions of normal human serum (NHS) and at a multiplicity of infection of 10.

### Neutrophil Killing Assays

For the killing assays, *S. pneumoniae* strains previously incubated in various concentrations of human sera obtained from healthy volunteers (diluted in PBS) at room temperature for 30 mins were added to fresh human neutrophils extracted from blood [81] in HBSS with divalent cations at an MOI of 1∶800. After 45 mins at 37°C, the numbers of surviving bacteria were calculated by plating serial dilutions, and the results expressed as a percentage of the inoculum CFU.

### ABC Transporter Phenotype Analysis

Sensitivity to oxidative stress and cation transport were studied by exposure of *S. pneumoniae* strains (10^6^ cfu) to 60 mM of paraquat (Sigma) [30] at 37°C for 20, 40 and 60 min. The proportion of survivors after the exposure was calculated by plating serial dilutions on blood agar plates. Zn^+2^ uptake was measured by a fluorescence assay as described by Bayle et al [42]. Bacteria (2×10^8^ CFU) grown to mid log phase in CDM were washed in PBS and incubated with 5 µM FluoZin-3 AM, (acetoxy methyl ester) cell permeant (Molecular Probes) for 30 min at room temperature. The bacteria were washed three times in PBS and then incubated for a further 30 min to allow complete de-esterification of intracellular acetoxymethyl FluoZin-3 esters. All the experiments were performed at 37°C under stirring conditions using a Photon Technology International Quanta Master I spectrofluorimeter. Upon the addition of 10 µM of ZnSO4 and excitation of the sample at 494 nm, fluorescence emission was recorded at 516 nm and the rate of zinc uptake (arbitrary unit sec^−1^) was calculated from the slope of the curve.

### ICP-MS Analysis

Total internal concentrations of metal ions was carried out by the highly sensitive ICP-MS analysis [82]. 5×10^8^ CFU of mid log phase bacteria grown in THY–chelex were washed extensively with chelex treated PBS and resuspended in 5 ml of 2% nitric acid. The bacteria were further lysed by sonication (3 pulses of 20 sec with a 20 sec cooling time) using a Soniprep 150 (Sanyo) ultrasonicator and filtered through 0.45 µ millipore filters to discard cellular debris. MilliQ water and PBS needed for dilution and washes were treated over night with Chelex 100. The ICP-MS analysis was carried out with a Varian ICP-MS instrument.

### Growth in Physiological Fluids

Replication of *S. pneumoniae* strains in freshly obtained human blood and frozen mouse bronchoalveolar lavage fluid (BALF) was determined by inoculating with 2×10^6^ CFU ml^−1^. After 4 h of growth at 37°C under CO_2_, serial dilutions were plated on to blood agar plates to enumerate the CFU.

### *In vivo* Studies

All animal experiments conformed to institutional and governmental guidelines and regulations. Outbred CD1 female white mice (Charles Rivers Breeders) weighing 18–22 g were used for animal infection experiments. For the pneumonia model mice were anaesthetized by inhalation of halothane (Zeneca) and inoculated IN (intra nasal) with an inoculum of 5×10^6^ CFU/mouse in 50 µl volume, and for the septicaemia model by IP (intra peritoneal) inoculation of 7×10^3^ CFU in 100 µl volume. Mixed infection experiments were used to calculate CIs (the ratio of mutant to wild-type strain recovered from the mice divided by the ratio of mutant to wild-type strain in the inoculum). Mice were sacrificed after 24°h (septicaemia model) or 48°h (pneumonia model), target organs recovered and homogenized in sterile PBS, before plating dilutions on non-selective and selective medium for calculation of the CI. For the nasopharygneal colonisation model, 10^7^ CFU of bacteria in 10 µl were administered by intranasal inoculation under halothane general anaesthesia, and nasal washes were obtained after various time points. Serial dilutions of the samples were plated onto Columbia blood agar plates containing optochin (50 µg ml^−1^) and/or gentamycin (5 µg ml^−1^) to differentiate pneumococcus from other contaminating streptococci and to enumerate CFU. To compare the course of disease between the Δ*lgt* and wild-type strains, groups of 10 mice were inoculated with 3×10^3^ CFU IP of either strain and closely observed over the next 14 days. Mice were sacrificed when they exhibited the following signs of disease: hunched posture, poor mobility, weight loss, coughing and tachypnoea.

### Statistical Analysis

All in vitro data use three or more samples per strain tested, and are representative of experiments repeated at least twice that gave similar results. Results for phenotype assays were compared between strains using Student’s t test or ANOVA. Experiments comparing the course of disease between the Δ*lgt* and wild-type strains were repeated twice, giving similar results, and the data analysed using the log rank method for survival.
